# Semantic and psychometric validation of the Brazilian Portuguese version (PASE-P) of the Psoriatic Arthritis Screening and Evaluation questionnaire

**DOI:** 10.1371/journal.pone.0205486

**Published:** 2018-10-11

**Authors:** Carolina Zorzanelli Costa, Claudia Goldenstein-Schainberg, Sueli Carneiro, José Joaquim Rodrigues, Ricardo Romiti, Thiago Bitar Martins Barros, Gladys Martins, Jamile Carneiro, Rachel Grynszpan, Ana Luisa Sampaio, Tânia Maria Silva Mendonça, Carlos Henrique Martins Silva, Abrar A. Qureshi, Rogerio de Melo Costa Pinto, Roberto Ranza

**Affiliations:** 1 Universidade Federal Uberlândia, Uberlândia, Brasil; 2 Faculdade de Medicina, Universidade de São Paulo, São Paulo, Brasil; 3 Hospital Universitário Clementino Fraga Filho, Universidade Federal do Rio de Janeiro, Rio de Janeiro, Brasil; 4 Hospital Universitário de Brasília, Brasília, Brasil; 5 Warren Alpert Medical School, Brown University, Providence, Rhode Island, United States of America; MGH Institute of Health Professions, UNITED STATES

## Abstract

PASE (Psoriatic Arthritis Screening and Evaluation) was developed in the English language to screen for inflammatory arthritis among patients with psoriasis. It is 15 item self administered questionnaire with a score from 15 to 75. A higher score indicates a greater risk for inflammatory joint disease. The purpose of this study was to translate, adapt and validate this questionnaire into Brazilian Portuguese (PASE-P). METHODS: 465 patients diagnosed with psoriasis (158 with psoriatic arthritis confirmed by a rheumatologist according to the CASPAR criteria and 307 without) were evaluated in dermatology clinics. We performed the analysis of semantic equivalence in eight steps. For psychometric equivalence, we evaluated the data quality, reliability, construct validity, well-known groups and discriminant characteristics of the items, as well as a ROC curve to determine optimal PASE-P cutoff points in case identification and their sensitivity / specificity. The final version presented excellent reproducibility (CCI = 0.97) and reliability (Cronbach’s alpha> 0.9). A cut-off point of 25 distinguished between patients with and without psoriatic arthritis, with sensitivity of 69.5 and specificity of 86.8. PASE-P proved to be culturally valid and reliable to screen for psoriatic arthritis in Brazilian patients with psoriasis.

## Introduction

Psoriatic arthritis (PsA) is a chronic inflammatory disease associated with psoriasis (Pso) [1,2], with reported prevalence varying from 6 to 42% and values of 30% in a Brazilian population [2–4]. PsA is difficult to diagnose in its early stages; however, it can progress, causing anatomical damage that impairs the functional capacity of patients [3,5,6].

There is agreement that the early diagnosis of PsA allows for the establishment of more effective treatments to avoid or minimize the physical and psychosocial impairment of patients [3,5,6].

Several studies have shown that PsA is not identified and treated in a timely manner in many cases [4]. There is a consensus on the need for strategies for the early identification of PsA among patients with Pso. One of the most effective strategies developed is the use of self-report questionnaires to detect patients at high-risk for inflammatory joint disease [7,8].

Among the specific questionnaires for screening for PsA, the most widely used are Psoriasis Epidemiology Screening Tool (PEST) [8], Toronto Psoriatic Arthritis Screen (ToPAS) [9] and Psoriatic Arthritis Screening and Evaluation (PASE) [10,11]. The PASE is the most commonly used tool for PsA screening in Pso clinics in our country. It was developed by a partnership between dermatologists and rheumatologists for application in dermatology clinics. It is a rapid test consisting of 15 items with a possible score ranging from 15 to 75; a higher score indicates a greater probability that the patient has PsA. Studies have shown that it is effective for identifying active PsA and satisfactorily discriminates inflammatory arthritis from osteoarthritis. [10,11]. Because it is applied to identify patients who require rheumatological evaluation, its screening sensitivity and specificity should be adapted to rheumatology unit availability. In this context, the continuous score allows adjustment of the cutoff value to yield a wider or more restricted referral range, which increases the specificity to assure rheumatological assessment in more severe cases [11]. Within this framework, the PASE is an important tool for the identification of PsA patients by facilitating timely referral to rheumatologists and adequate treatment.

The PASE was developed in American English and translated into more than 20 languages. For use in Brazil, the PASE still requires translation, cross-cultural adaptation and assessment of its psychometric properties according to international standards to ensure the maintenance of the concepts established in the original instrument [12]. The aim of the present study was to translate, cross-culturally adapt, and validate a Brazilian Portuguese version of PASE (PASE-P).

## Methods

### Study and participants

We conducted a multi-center, cross-sectional study for the translation and cross-cultural adaptation of PASE. This study was conducted at the dermatology outpatient clinics of four Brazilian university centers: Federal University of Uberlândia (Universidade Federal de Uberlândia, UFU), University of São Paulo (Universidade de São Paulo, USP), University of Brasília (Universidade de Brasília, UnB) and Federal University of Rio de Janeiro (Universidade Federal do Rio de Janeiro, UFRJ). The research ethics committees of each participating center (**Ethics Committee for Research Involving Human Beings of the Federal University of Uberlandia, Ethics Committee for Research Involving Human Beings of the Health Sciences School of the University of Brasilia, Ethics Committee for Research Involving Human Beings** of the Clementino Fraga Filho University Hospital—Federal University of Rio de Janeiro and **Ethics Committee for Research Involving Human Beings of the** University of Sao Paulo) granted approval, and the PASE copyright owners granted authorization. We used the methods formulated by Eremenco et al. [12]. Participants were patients aged > 18 years old who signed an informed consent form, could read and understand the PASE items, and were diagnosed with Pso by a dermatologist.

All patients with Pso were assessed by a rheumatologist to investigate the presence of rheumatic diseases, including PsA, which was classified according to the CASPAR criteria [1]. Patients with incorrectly completed questionnaires, defined as patients who failed to respond to two or more PASE items, were excluded from the study.

### Sample size

In the assessment of semantic equivalence, we tested the translated version of PASE with 20 and 40 representatives of the target population at two different time-points, respectively [13].

In the assessment of measurement equivalence, we chose to calculate and design the sample to be representative of the studied population. Therefore, we considered the population size at the four participating institutions (n = 6,500), the prevalence of the outcomes (considered 50% for multiple outcomes), 5% error, and a sample design effect of 2. The sample size was estimated as 363 participants, but to compensate for possible losses and refusals, it was multiplied by 1.2, resulting in a minimum of 436 participants.

### Instrument

#### Psoriatic Arthritis Screening and Evaluation (PASE)

The PASE was developed by Husni et al. as a tool to identify the Pso patients most likely to have PsA. [10] It comprises 15 items answered on a 5-point Likert scale with the responses “Strongly disagree”, “Disagree”, “Neutral”, “Agree” and “Strongly agree”. The items are divided into two subscales: Symptoms, with 7 items (score 7 to 35), and Function, with 8 items (score 8 to 40). The total PASE score is the sum of the two subscale scores, ranging from 15 to 75. The pilot study showed that higher scores indicate greater odds of having PsA (with 82% sensitivity and 73% specificity) vs. osteoarthritis [10]. The PASE validation study confirmed its ability to distinguish PsA from non-PsA in a larger population [11]. The total PASE scores ranged from 15 to 74, and a value of 44 was set as the optimal cutoff point to distinguish PsA from non-PsA, yielding 76% sensitivity and specificity. The test-retest reliability of the PASE and its sensitivity to change after systemic treatment were also evaluated, which yielded statistically significant results [11].

### Procedures (Fig 1)

**Fig 1:**
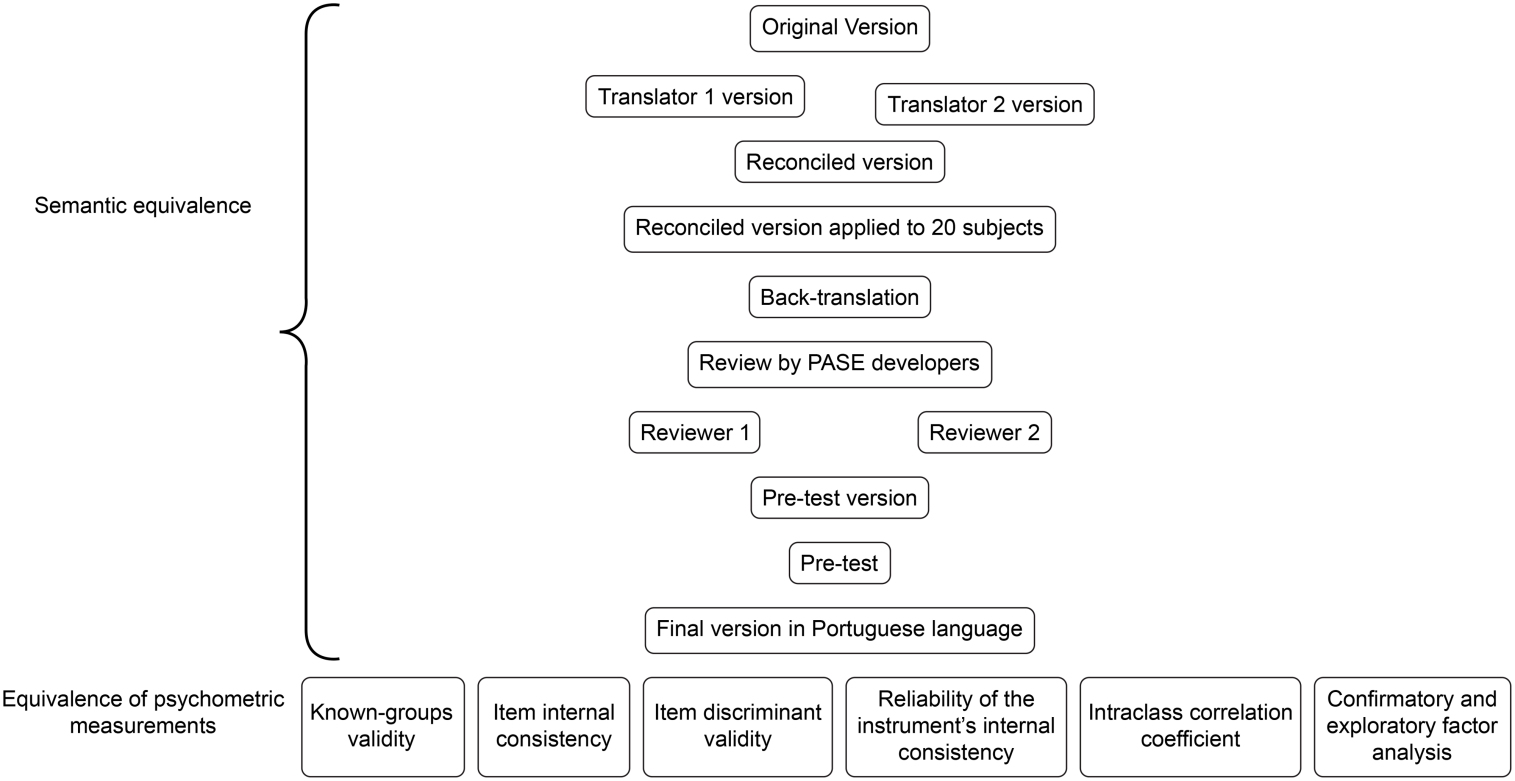
Semantic and psychometric equivalence of PASE-P.

The translation and cross-cultural adaptation of PASE included an assessment of its semantic equivalence and psychometric properties. In both stages of the study, the participants self-administered the PASE-P and a questionnaire for sociodemographic and clinical data (age, gender, educational level, time since diagnosis of Pso and associated diseases).

### Assessment of sematic equivalence

**Fig 2:**
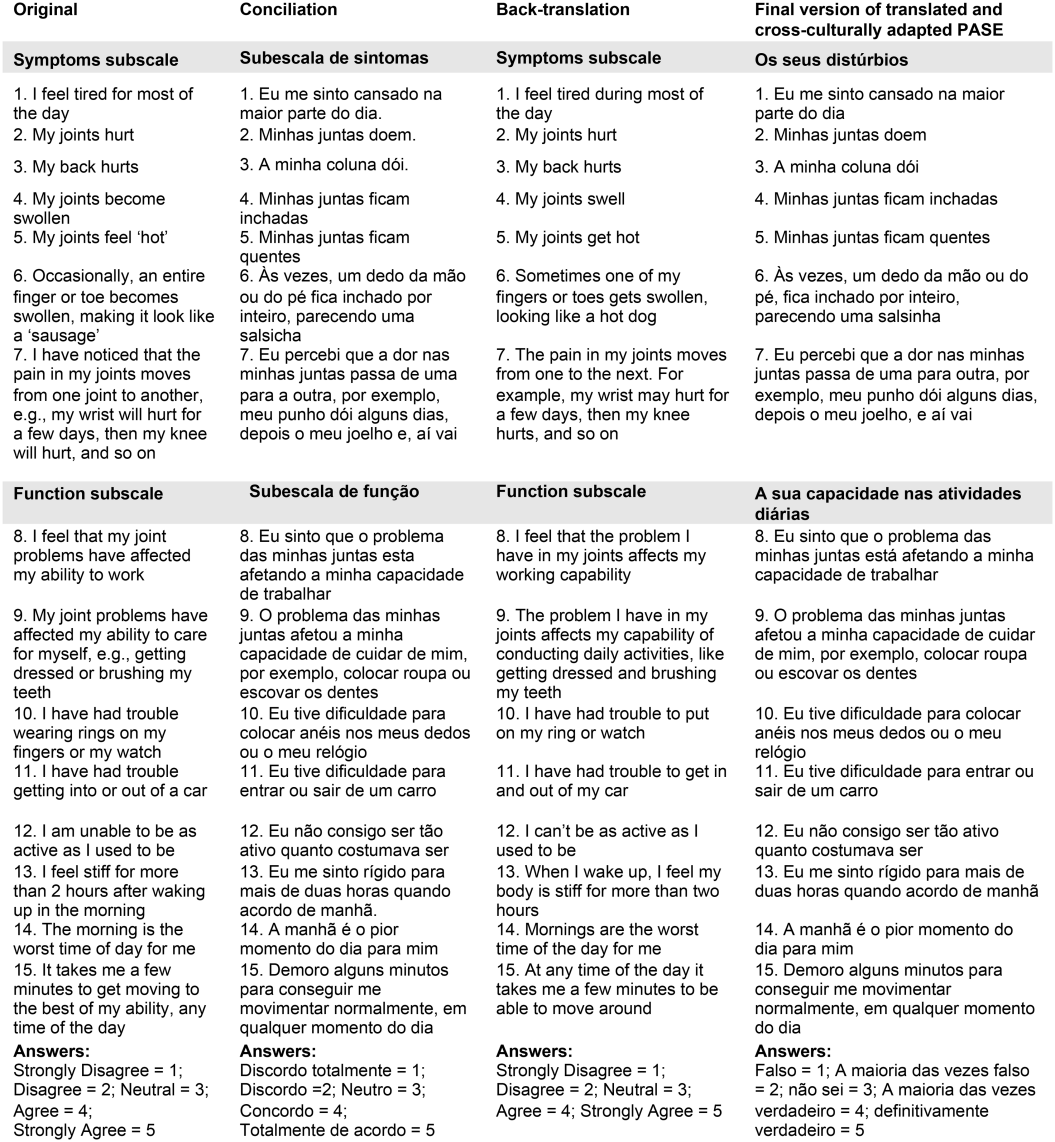
Conciliation, back-translation and final version of translated and cross-culturally adapted PASE.

This stage was performed in eight steps (Fig 1). The first step, initial translation, was performed by two professional translators who are native Brazilian Portuguese speakers, resulting in two independent translations. The second step, reconciliation of the two translations, was performed by two native Brazilian Portuguese speakers who are fluent in English, have medical knowledge, and did not participate in the previous step. In step three, back-translation, the reconciled version was translated back to the original language by a professional who is a native English speaker, fluent in Brazilian Portuguese, who did not participate in the earlier steps, and had no knowledge of the original instrument. Step four consisted of a cognitive face-to-face interview, in which the instrument was applied by one of the researchers at the Federal University of Uberlândia to 20 subjects to determine their understanding of the items. The subjects had difficulty understanding the headings of the two subscales and the headings of the response categories. Fig 2 shows the adaptations that were made.

In step five, the back-translated version was sent to the PASE developers’ linguistic team for review. In step six, a report was sent to two bilingual (English and Brazilian Portuguese) reviewers, who independently analyzed the forward translations, the reconciled version, the back-translation, the reports of each step, the original version, and the comments made by the developers of the original instrument. Step seven consisted of preparing a version of the PASE for the pre-test. Step eight consisted of PASE pre-testing, during which it was applied to 40 patients who were predominantly female (70%) and had variable educational levels (20% incomplete elementary school, 30% complete elementary school, 36% complete secondary school, and 14% complete higher education). The test subjects did not report any difficulty understanding the instrument.

### Assessment of the equivalence of psychometric measurements

A total of 465 patients diagnosed with Pso participated in the assessment stage of the psychometric properties of PASE. In this stage, we assessed the quality, reliability, and validity of the data.

The analysis of data quality included verification of the missing data (under 20%) [14] and the floor and ceiling effects (over 10%) [14] for each PASE item.

The assessment of the reliability of the PASE included an analysis of the internal consistency of the items, the reliability of the internal consistency of the instrument, and the test-retest reliability (second application of the instrument using the self-applied method by the same examiner to a random sample of 30 patients two weeks after the first application).

The assessment of the validity of PASE included an analysis of item discriminant validity, known-groups validity, and construct validity.

### Statistical analysis

A descriptive analysis was used for the sociodemographic and clinical characterization of the participants. The floor and ceiling effects of PASE were calculated as the proportion of patients with the lowest and highest scores on the scale, respectively.

The reliability of the instrument was assessed by means of the correlation between items and the corresponding subscale and Cronbach’s alpha and intraclass correlation coefficient (ICC). Values over 0.7 and 0.75, respectively, were considered satisfactory [14,15].

The construct validity was evaluated by the transcultural translation and adaptation (transcultural validity), validity of known groups and structural validity. The structural validity was evaluated using confirmatory factorial analysis. For this purpose, the database of respondents was randomly divided into two equal groups (www.randomizer.com). In one group, a CFA was performed assuming that all PASE items had a factorial load of only one factor, which represents a structural equation modeling confirmatory strategy used to explain the relationship between items and the general factor or construct associated with PsA [16]. Weighted least squares with adjustments for the mean and variance (WLSMV) was the estimation method used. The fit indices analyzed were as follows: comparative fit index (CFI; ideal when > 0.90), root mean square error approximation (RMSEA; **<** 0.08 adequate fit, < 0.06 satisfactory fit), and the Tucker-Lewis index (TLI; ideal when > 0.90) [17,18].

The data corresponding to the second group were used for Exploratory Factor Analysis (EFA) by means of parallel analysis (PA). We applied polychoric correlations and unweighted least squares as a method to extract ordinal categorical data, with oblimin rotation for factor rotation and interpretation of factor loading.

Spearman’s correlation coefficient was used to calculate the item discriminant validity to establish whether the items in each factor exhibited satisfactory correlation. Patients with Pso with or without PsA were compared in the analysis of known-groups validity by means of the Mann-Whitney U test.

A ROC (receiver operating characteristic) curve was plotted to establish the cutoff point of the total PASE scores and its sensitivity and specificity, which indicate higher odds of arthritis associated with Pso.

The data were analyzed using statistical software SPSS 20.0, Factor 8.0, MPLUS 6 and MedCalc. The significance level was set to 5%.

## Results

### Translation and cross-cultural adaptation

#### Assessment of sematic equivalence

Cognitive interviews showed no difficult to understand, irrelevant or offensive questions and that transcultural translation and adaptation had resulted in the respondents’ full understanding of the meaning of each item. Thus, the semantic and conceptual equivalence of each item of the instrument was confirmed. Steps one through five were reconciled by participants. Regarding step six, Fig 2 shows that changes were necessary in the headings of the two subscales and in the description of the response options. The subscale heading *“symptoms subscale”* was changed from *“**subescala de sintomas**”* to *“**os seus distúrbios**”*. The subscale heading *“function subscale”* was changed from *“**subescala de função**”* to *“**a sua capacidade nas atividades diárias**”*. The answer terms *“strongly disagree”*, *“disagree”*, *“neutral”*, *“agree”* and *“strongly agree”* were changed from *“**discordo totalmente**”*, *“**discordo**”*, *“**neutro**”*, *“**concordo*” and *“**totalmente de acordo**”* to *“**falso**”*, *“**a maioria das vezes falso**”*, *“**não sei**”*, “*a maioria das vezes verdadeiro**”* and “*definitivamente verdadeiro**”*, respectively. After the pre-test, conducted in step eight, the final version of the instrument was created.

### Equivalence of psychometric measurements

A total of 465 patients with Pso completed the study with an average time of 9.8 (± 2.4) minutes. No participants were excluded from analysis because no assessments with missing data on two or more questions were found. The participants had an average age of 48.8 ± 15.7 years old, and 50% were female. The average duration of Pso was 15.5 ± 11.8 years. The characteristics of the study population and diagnosed rheumatic conditions are described in Table 1. PASE-P distinguished between participants with (n = 158) and without (n = 307) PsA, with average scores of 37.8 ± 17.2 vs. 22.7 ± 11.5, respectively (p < 0.001). Table 2 describes the various cutoff points we tested and the corresponding sensitivity (SE) and specificity (SP). Interestingly, a cutoff of 25 was associated with a 0.695 SE (95% confidence interval—CI 0.592, 0.785) and a 0.868 SP (95% CI 0.816, 0.91). PsA patients without any systemic treatment (n = 42) exhibited the highest PASE-P scores (mean 47.5 ± 14.1). This group, compared to non-PsA, had a cutoff point of 38 and an SE of 0.786 and an SP of 0.876 (area under the curve—AUC: 0.908, 95% CI 0.868–0.948). A sub-analysis of PsA vs. osteoarthritis (OA) yielded scores of 37.8 vs. 24.4 ± 14.3 (p < 0.001) and a cutoff of 22 with an SE of 0.785 and an SP of 0.697 (AUC 0.881, 95% CI 0.818–0.945). The PASE-P exhibited high internal consistency (Cronbach’s alpha 0.926), and the test-retest ICC was 0.974.

**Table 1:** Characteristics of study participants with or without a diagnosis of PsA.

	PsA		
Variables	Present	Absent	p_value_
	(n = 158)	(n = 307)	
Age, m (SD)	50.2 (14.8)	48.2 (14.7)	0.02
Female, n (%)	93 (58.9)	140 (45.6)	0.348^c^
OA^a^, n (%)	41 (26)	33 (10.8)	0.000^c^
Fibro^b^, n (%)	9 (5.7)	24 (7.8)	0.199^c^
OA and Fibro, n (%)	10 (6.3)	10 (3.2)	0.061^c^
PASE, m (P25-P75)	33 (21–47)	18 (19–29)	< 0.05^d^

^a^OA = Osteoarthritis.

^b^Fibro = Fibromyalgia.

^c^Binomial test for two proportions.

^d^Mann-Whitney.

**Table 2:** PsA vs, non-PsA—PASE-P sensitivity and specificity according to cutoff point.

Cutoff point	Sensitivity	95% CI	Specificity	95% CI
19	90.5	82.8–95.6	70.0	63.5–76.0
20	86.3	77.7–92.5	73.2	66.8–78.9
21	81.1	71.7–88.4	75.5	69.2–81.0
22	80.0	70.5–87.5	79.6	73.6–84.7
23	76.9	67.1–84.9	81.8	76.1–86.7
24	71.6	61.4–80.4	84.6	79.1–89.1
25	69.5	59.2–78.5	86.8	81.6–91.0
26	66.3	55.9–75.7	88.2	83.2–92.1
27	63.2	52.6–72.8	88.7	83.7–92.5
28	62.1	51.6–71.9	89.6	84.7–93.3
29	61.1	50.5–70.9	90.0	85.3–93.6
30	57.9	47.3–68.0	91.8	87.4–95.1
31	54.8	44.2–65.0	92.3	87.9–95.4
32	52.6	42.1–63.0	94.1	90.1–96.8
33	50.5	40.1–60.9	94.1	90.1–96.8
35	50.5	40.1–60.9	95.9	92.4–98.1
36	49.5	39.1–59.9	95.9	92.4–98.1
37	49.5	39.1–59.9	96.8	93.6–98.7
38	46.3	36.0–56.8	97.3	94.2–99.0
39	44.2	34.0–54.8	97.7	94.8–99.3
43	43.2	33.0–53.7	97.7	94.8–99.3
45	41.1	31.1–51.6	98.2	95.4–99.5

The construct validity was confirmed by means of CFA and EFA. In the CFA, the fit indices showed that the model was consistent with the data (CFI = 0.98; TLI = 0.97; RMSEA = 0.07; WRMR = 0.94). The factor loading was greater than 0.7 with residuals close to zero. The interpretability of the parameters, assessed based on the significance of standard error, was satisfactory. In the EFA, we confirmed that the data matrix was adequate for factoring by means of polychoric correlations over 0.3; the Kaiser-Meyer-Olkin (KMO) index was 0.92, and the results of Bartlett’s sphericity test were significant (p < 0.0001). We extracted two factors by means of EFA; the first factor explained 63% of the variance, and the second factor explained 7%. The ratio of variance explanation between the factors was 9, with a correlation of 0.6.

On the assessment of item discriminant validity, our success rate was 100% for the correlation among the items in each subscale and the domain represented by each subscale (symptoms: 0.42; function: 0.41–0.72). The median and the 25^th^ and 75^th^ percentiles of the total PASE score were significantly higher (p < 0.05) for the patients with PsA compared to the patients without this condition (31 (16;47) vs. 21 (15;37)), thus confirming the known-groups validity.

## Discussion

Our study shows that the Brazilian Portuguese version of PASE (PASE-P) is valid, reliable, and reproducible for use in Brazilian dermatology clinical practice. The changes made in the headings of both subscales and the text of the answer options were crucial to ensure the cross-cultural validity of the instrument. The cognitive interview during the pre-test confirmed the semantic and conceptual equivalence of each item of the instrument.

Considering that, in most patients, Pso affects the skin approximately one decade before it affects the joints, PASE-P is a relevant screening tool, particularly for dermatologists who care for patients with Pso [12,13]. Similar to versions in other languages [14,15], the PASE-P exhibited excellent reliability. The quality of PASE-P was also confirmed by the lack of floor and ceiling effects on total score. This shows that the study population, originating from outpatient care centers, exhibited different disease activity levels and that PASE scores vary with disease activity.

The psychometric quality of the instrument, maintained by the translation and cross-cultural adaptation process, was also confirmed by the construct, discriminant, and known-groups validity. CFA and EFA showed that the model had adequate fit. These analyses also confirmed the construct validity of the PASE-P by showing that its two subscales assess one single dimension. The methodological rigor used to ensure semantic and measurement equivalence guaranteed PASE-P robustness as a self-administered screening instrument.

In this study, 25 was one of the best cutoff points to distinguish between patients with and without PsA, with an acceptable SE of 0.695 and a good SP of 0.868. In the validation study of the original PASE, the best cutoff point for this purpose was higher, 44, with an SE of 76% and an SP of 76%, close to those of the present study [11]. In a study to validate an Argentinian PASE version, the best cutoff point to distinguish between patients with and without PsA was also different, 34, but SE and SP were once again similar, at 76% and 74.4%, respectively [19]. These findings demonstrate the ability of PASE to distinguish between patients with and without PsA. Furthermore, they show that the ideal score may vary as a function of the population to which the instrument is applied. The flexibility of the scores suggests that the cutoff point can be chosen according to the availability of access to rheumatologic assessment. Low scores with high SE and low SP would be preferred in units with a high availability of rheumatologic assessment and in combined clinical facilities. Conversely, high scores, which favor SP, would be preferred in settings with limited access to rheumatologists.

The main flaw of the PASE is its poor ability to distinguish between PsA and OA because OA is a frequent condition in the age group of PsA patients [20]. In our case series, OA was diagnosed in 20.2% of the patients, exhibiting partial overlap with PsA. The PASE-P scores for patients with PsA (with or without associated OA) vs. OA were significantly different (37.8 vs. 24.4 ± 14.3; p < 0.001). However, the cutoff point had to be reduced to 22, with satisfactory SE (0.785) but relatively low SP (0.697). This confirms data from the Contest study [20], which found an SE of 74.5% but an SP of only 38.5% for PASE. In contrast, in PsA patients not on DMARD treatment, the cutoff point was 38 with a SE of 0.786 and a SP of 0.876 (AUC: 0.908, 95% CI 0.868–0.948). In these patients PASE-P showed to be more discriminatory as in the original validation study [11].

The main limitation of the present study is its cross-sectional nature. Additionally, it assessed a heterogeneous population of patients that included cases of Pso under systemic treatment for the dermatological disease and patients who were already diagnosed and treated for PsA. These treatments may have influenced the establishment of the cutoff point for PASE-P to distinguish patients with or without arthritis, as occurred in the original PASE validation study [11]. This was confirmed as follows: the group that did not receive any systemic treatment had the highest cutoff point in the present study (38, with an SE of 0.786 and an SP of 0.876). These considerations reinforce the need to use this instrument with careful assessment of patient characteristics and the best cutoff point.

In addition to reducing PsA detection time, the application of a well-designed, valid and reliable screening tool may help determine the prevalence of PsA in a given population of Pso patients [4,11].

The Brazilian Portuguese version of PASE (PASE-P) is valid and reliable for use in dermatological clinical facilities to identify Pso patients with higher odds of developing PsA.
